# Exploring the Global and Regional Factors Influencing the Density of *Trachurus japonicus* in the South China Sea

**DOI:** 10.3390/biology14070895

**Published:** 2025-07-21

**Authors:** Mingshuai Sun, Yaquan Li, Zuozhi Chen, Youwei Xu, Yutao Yang, Yan Zhang, Yalan Peng, Haoda Zhou

**Affiliations:** 1 Key Laboratory for Sustainable Utilization of Open-Sea Fishery, Ministry of Agriculture and Rural Affairs, South China Sea Fisheries Research Institute, Chinese Academy of Fishery Sciences, Guangzhou 510300, China; sunmingshuai@scsfri.ac.cn (M.S.); xuyouwei@scsfri.ac.cn (Y.X.);; 2 Taishan Nuclear Power Joint Venture Company Limited, Taishan 529200, China; tlbbweixin1@163.com (Y.L.);; 3 Zhuhai Marine Center, Ministry of Natural Resources, Key Laboratory of Marine Environmental Survey Technology and Application, Zhuhai 519015, China

**Keywords:** *Trachurus japonicus*, machine learning, causal inference, fisheries management, ozone concentration, solar flux index, South China Sea

## Abstract

In this cross-disciplinary investigation, we uncover a suite of previously unexamined factors and their intricate interplay that hold causal relationships with the distribution of *Trachurus japonicus* in the northern reaches of the South China Sea, thereby extending the existing research paradigms. Leveraging advanced machine learning algorithms and causal inference, our robust experimental design uncovered nine key global and regional factors affecting the distribution of *T. japonicus* density. A robust experimental design identified nine key factors significantly influencing this density: mean sea-level pressure (msl-0, msl-4), surface pressure (sp-0, sp-4), Summit ozone concentration (Ozone_sum), F10.7 solar flux index (F10.7_index), nitrate concentration at 20 m depth (N3M20), sonar-detected effective vertical range beneath the surface (Height), and survey month (Month). Crucially, stable causal relationships were identified among Ozone_sum, F10.7_index, Height, and N3M20. Variations in Ozone_sum likely impact surface UV radiation levels, influencing plankton dynamics (a primary food source) and potentially larval/juvenile fish survival. The F10.7_index, reflecting solar activity, may affect geomagnetic fields, potentially influencing the migration and orientation behavior of *T. japonicus*. N3M20 directly modulates primary productivity by limiting phytoplankton growth, thereby shaping the availability and distribution of prey organisms throughout the food web. Height defines the vertical habitat range acoustically detectable, intrinsically linking directly to the vertical distribution and availability of the fish stock itself. Surface pressures (msl-0/sp-0) and their lagged effects (msl-4/sp-4) significantly influence sea surface temperature profiles, ocean currents, and stratification, all critical determinants of suitable habitats and prey aggregation. The strong influence of Month predominantly reflects seasonal changes in water temperature, reproductive cycles, and associated shifts in nutrient supply and plankton blooms. Rigorous robustness checks (Data Subset and Random Common Cause Refutation) confirmed the reliability and consistency of these causal findings. This elucidation of the distinct biological and physical pathways linking these diverse factors leading to *T. japonicus* density provides a significantly improved foundation for predicting distribution patterns globally and offers concrete scientific insights for sustainable fishery management strategies.

## Simple Summary

Preventing the decline of *Trachurus japonicus*—a threatened fish vital to South China Sea fisheries—remains complex even as environmental pressures intensify. Using a machine learning analysis of oceanic and climate data (2014–2020), we identified nine key factors controlling the fish’s distribution, including atmospheric pressure, ozone levels, solar activity, and ocean nutrients. Significantly, we found consistent cause-and-effect links between four factors: ozone concentrations measured in Greenland, solar radiation levels, sonar-detected ocean layering depths, and nitrate concentrations 20 m below the surface. While these global patterns strongly drove fish movements (likely by altering light, nutrients, or navigation), human activities like shipping showed unexpectedly weak causal impacts—though their role remains critical elsewhere. We conclude that natural factors like Arctic ozone changes disproportionately affect tropical fish stocks, which may challenge local conservation efforts amid global environmental shifts.

## 1. Introduction

*Trachurus japonicus*, widely known as the Japanese jack mackerel, stands as a cornerstone species in the marine ecosystems of the Northern South China Sea, contributing significantly to regional fisheries and food security [1]. In China, it ranks as the nation’s second-largest marine fishery catch after hairtail (*Trichiurus lepturus*), with 2022 landings reaching 395,862 tons (*China Fishery Statistical Yearbook*). As a vital economic resource for southeastern coastal provinces, particularly Fujian, it is primarily harvested through light seine nets and trawl fisheries. However, this species faces significant pressures: landings have exhibited a concerning overall decline in recent years, positioning it as a critical indicator of ecosystem health and anthropogenic impacts. Its dramatic population fluctuations recently prompted its inclusion on the IUCN Red List of near-threatened (NT) species [2,3,4,5]. This species exemplifies a model organism for studying multifactorial marine ecosystem dynamics due to three key attributes: its inherent sensitivity to biotic/abiotic variables (temperature, salinity, food availability), its high fisheries exploitation pressure, and its documented population decline mirroring regional environmental changes. Consequently, its spatial and density patterns serve as a critical barometer for marine ecosystem resilience amid escalating global environmental change and human activities [6,7].

Globally, fisheries face unprecedented pressures from climate change, with rising sea temperatures altering species distributions [8], while regional anthropogenic stressors, such as overfishing and habitat degradation in the South China Sea [9], compound these challenges. Despite broad recognition of these threats, mechanistic studies linking global atmospheric dynamics to localized fish population responses remain nascent.

Current research prioritizes linear relationships, like temperature vs. biomass [5], or singular stressors [1], neglecting the synergistic interplay of climatic, oceanic, and anthropogenic drivers. Crucially, the causal pathways through which high-latitude atmospheric factors—such as Arctic ozone concentrations—influence subtropical fisheries are unidentified [10,11], representing a critical knowledge gap for predictive modeling.

Here, we bridge this gap by integrating machine learning (ML) and causal inference to decode multifactorial controls on *T. japonicus* density from 2014 to 2020. Our study examined complex non-linear associations between fish distribution and 309 feature variables, spanning natural environmental factors, anthropogenic pressures, spatiotemporal parameters, global climate indicators, and a controlled random factor. The use of SHAP (SHapley Additive exPlanations) not only enabled us to decipher the contributions of individual features in our predictive models but also allowed for the exploration of interactions among these factors, thereby offering enhanced interpretability and accountability [12,13,14]. On the other hand, the inclusion of causal analysis was pivotal for elucidating the underlying cause-and-effect relationships among these variables. Specifically, rigorous refutation tests using Data Subset Refutation and the Random Common Cause Refutation methods were employed in causal inference, providing a more comprehensive understanding of the determinants affecting *T. japonicus* [15,16,17,18,19].

Among these variables, global climatic factors, such as the El Niño index, along with regional climatic variables, including sea surface temperature, atmospheric pressure, and wind speed, exert a pronounced influence on fish distribution. In particular, their lag effects are considered to be vital in driving changes in fish populations [20,21]. In addition, environmental factors, namely depth, latitude, longitude, and nutrients (including phosphates, nitrates, nitrites, and silicates at depths of 0 m, 10 m, and 20 m), have been identified as significant influences on fish distribution. For instance, the nitrate concentration at a depth of 20 m (N3M20) and the vertical range beneath the sea surface detected by sonar (Height) are critical factors [22,23].

Anthropogenic factors like maritime traffic and fishing operations also critically influence fish distribution [24]. While this study did not indicate a strong causal relationship between these anthropogenic factors and the distribution of *T. japonicus*, the potential influence of such factors is undeniable and demands continued attention.

The sea surface partial pressure of CO_2_ and other global carbon sink-related factors, though not showing a strong causality in this research, are important potential factors that should be paid continuous attention, considering the significant role of marine carbon sinks in the global carbon cycle and climate change [25].

Our study identified the top features that significantly impact the distribution of *T. japonicus* density based on fishery acoustic data and catch data, including ozone (O_3_), F10.7 solar flux index, and nitrate concentration at a depth of 20 m. The insights gained from a comprehensive understanding of these influential factors and their intricate interactions can provide a scientific basis for the resource management of *T. japonicus* in the northern South China Sea. Moreover, it will contribute to the health of the marine ecosystem and the sustainable utilization of marine biological resources.

## 2. Materials and Methods

We performed a comprehensive analysis comparing the performance of optimized Random Forest [26] and XGBoost models [27], and investigated the causal relationships between selected features and fish density. Our methodology predominantly involved three steps: a model performance comparison, SHAP value analysis, and causal analysis using the DoWhy and EconML libraries [28,29,30].

### 2.1. Data Preparation

The experimental data primarily encompassed data collection and variable arrangement, which included the study area, actual samples, and global data.

**Figure 1:**
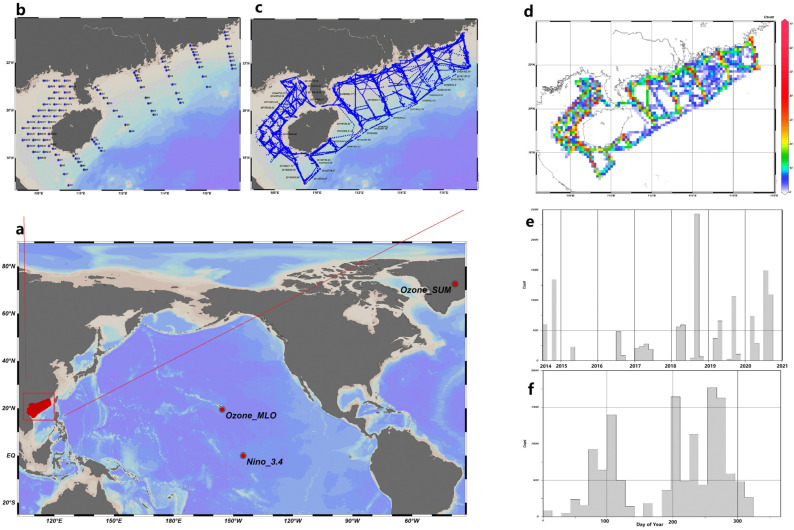
Spatial and temporal distributions of samples in the study: (**a**) Sample collection sites include regions such as the South China Sea, Greenland, and Hawaii. (**b**) Collection points for catch data, nutrient salts, and CTD (Conductivity, Temperature, and Depth) data were used. The discrete data were interpolated using Kriging interpolation. (**c**) Data points for hydroacoustics, climate, sea surface carbon dioxide partial pressure, AIS (Automatic Identification System) fishing, and the interpolated catch, nutrient salts, and CTD samples. (**d**) The spatiotemporal distribution of the sample points, where red indicates a high density of points and blue indicates a sparse distribution. (**e**) Distribution of monthly sample quantities from 2014 to 2020. In addition to the aforementioned spatial variables, there were also global variables that were sampled over time, such as geomagnetic activity, solar phenomena, and spatially integrated sea–air CO_2_ flux in grams of carbon per year (gC/yr). (**f**) A comprehensive distribution of the sample set in a date sequence (1–365). (Drawn by ODV [31]).

This study focused on the continental shelf of the northern South China Sea (16.5–23.5° N, 107.0–117.5° E; Figure 1), a critical traditional fishing ground sustaining China’s pelagic fisheries. This region spans a shallow plateau averaging < 200 m depth, where seasonal monsoons drive complex hydrographic dynamics (e.g., cyclonic gyres, coastal upwelling) and nutrient fluxes.

Actual samples, such as fishery acoustic data, catch data, water quality, and environmental information, were collected and acquired following operational guidelines. Water temperature, salinity, and depth were measured using AML Plus X CTD Profiler, and data on nutrients and transparency were also gathered. The surveys were conducted over twelve voyages from 2014 to 2020, specifically during the following periods: July–August 2014, October–November 2014, April–May 2015, July–August 2016, January–March 2017, April–June 2017, March–April 2018, August–October 2018, March–April 2019, August–October 2019, March–April 2020, and July–September 2020. The echosounder was calibrated at the start of each survey. All data, including the distribution of *T. japonicus* density based on fishery acoustic data and catch data [32,33], are explained in the Supplementary Materials.

Global data included climate data with lag effects, El Niño indices, ozone from SUM and MLO stations, data related to the Earth’s magnetic field, sea surface carbon dioxide data, and statistics on vessels at various distances based on AIS data. Samples were extracted from global data according to the survey time and area. All data were arranged in order of sample number, forming variables with a unified numbering order. All variables correspond one-to-one in each numbered sample.

The target variable was the population density of *T. japonicus*, obtained by converting and analyzing acoustic survey data. There were a total of 309 feature variables. All variables are explained in the Supplementary Materials.

### 2.2. Model Performance Comparison

The first stage of our investigation primarily focused on assessing and comparing the performances of two machine learning models, namely Random Forest [26] and XGBoost [27], in predicting fish density based on a set of selected features. The primary aim of this comparison was to ascertain the most appropriate model for our analysis, thereby laying the groundwork for subsequent investigations, such as feature importance and causal analysis. This stage was subdivided into three sections: model selection and hyperparameter optimization, model prediction and evaluation, and model performance comparison.

(1) Model Selection and Hyperparameter Optimization

Prior to comparing model performances, we first compared linear and non-linear models. Linear models were constructed using Linear Regression and Lasso Regression, while non-linear models employed XGBoost and Random Forest. The comparison revealed that non-linear models outperformed linear ones, leading us to select XGBoost and Random Forest to construct non-linear models. We then conducted an in-depth hyperparameter tuning of the Random Forest and XGBoost algorithms using grid search methods [34]. This step involved training models on the training dataset using a range of hyperparameter value combinations and evaluating their performances via cross-validation [35]. The optimal model for each algorithm was determined based on the lowest mean square error (MSE) [36] during the cross-validation process.

(2) Model Prediction and Evaluation

Upon identifying the best models, we evaluated their performance on the test dataset. The test dataset was separate from the training dataset and was not used during model training or hyperparameter tuning, ensuring an unbiased evaluation of each model’s predictive capability [36]. We used two established regression performance metrics, mean square error (MSE) [37] and R-square (R^2^) [38], to quantify the performance of the optimized Random Forest and XGBoost models.

(3) Model Performance Comparison

By comparing the MSE and R^2^ scores of the optimized Random Forest and XGBoost models on the test dataset, we identified the algorithm that exhibited superior predictive performance in estimating fish density based on the selected features. This comparison allowed us to select the model best suited for further analysis.

### 2.3. SHAP Value Analysis

During the second stage of our investigation, we implemented a SHAP (SHapley Additive exPlanations) value analysis to interpret the predictions of the selected model, identify the most influential features, and study the interactions between features [39,40]. This stage was divided into three sections: the principles of SHAP values, interaction effects, and the SHAP value analysis in our study.

(1) Principles of SHAP Values

SHAP values offer a consistent and locally accurate method to explain the output of any machine learning model [40]. The SHAP value of a feature is calculated by averaging the marginal contributions of the feature across all possible feature combinations [39].

(2) Interaction Effects

Moreover, SHAP values can be employed to detect and quantify interaction effects between features. Interaction effects occur when the combined impact of two or more features on a model’s prediction is not simply the sum of their individual contributions [39]. For instance, in our context, the impact of water temperature and oxygen level on fish density may not be merely additive, indicating the presence of interaction effects.

(3) SHAP Value Analysis in Our Study

After computing the SHAP values for each feature, we ranked them to identify the most influential features in predicting fish density. We also created SHAP plots to visualize the distribution of SHAP values for each feature and analyzed SHAP interaction values to inspect potential interaction effects between feature pairs. This step provided us with a comprehensive understanding of how individual features and their interactions contribute to the prediction of fish density.

### 2.4. Causal Inference

The methodological steps outlined in this study for causal inference begin with the formulation of an hypothesis, followed by data collection aligned with the research question. Selecting appropriate statistical methods is critical, with options ranging from regression analysis to complex machine learning algorithms like XGBoost, as highlighted in the Supplementary Materials. Controlling for potential confounders and conducting statistical tests assessed causal relationships’ strength and significance. The process concluded with a thorough interpretation of the results, considering any limitations and conducting a validation through robustness checks to establish credible causal inferences in ecological research.

We utilized the DoWhy and EconML libraries [28,29] to perform a causal analysis, studying the causal relationships between the most influential features identified via the SHAP analysis and fish density.

(1) Causal Inference

The principles of causal inference involve discerning true causative relationships from mere associations in ecological studies. This distinction is paramount, with emphasis on the counterfactual theory, where causation is understood by comparing observed outcomes with hypothetical scenarios in the absence of the treatment. The approach mandates robust methodologies to hypothesize and statistically test causal relationships, focusing beyond correlation to uncover underlying ecological dynamics [18,41].

Causal inference hinges on several preconditions: clearly defined hypotheses articulating cause–effect relationships, suitable and sufficient data to support or refute causal links, and ensuring the cause precedes the effect in time. Importantly, controlling for confounders is emphasized to avoid biased results. This is vital in ecological research, where numerous variables can influence outcomes, and hence a comprehensive understanding and adjustment for these confounders is necessary for valid causal conclusions [15,17].

(2) Causal Inference Evaluation

The final stage of the causal analysis involved evaluating the robustness of our causal estimates. We applied two key robustness checks, namely the Data Subset Refutation and the Random Common Cause Refutation.

The Data Subset Refutation involves using a subset of data to re-estimate the causal effect. If our original causal estimation is reliable and robust, the causal effect derived from the data subset should not deviate significantly from the original effect [30]. This approach provides a means of assessing the robustness of our causal findings across different subsets of data.

The Random Common Cause Refutation involves the introduction of an additional common cause (a confounder), which is independent of the existing covariates and treatment effect, to the data, followed by a re-estimation of the causal effect. Should our original causal estimation hold validity, the introduction of a random common cause should not significantly impact the causal estimate [30]. This method aids in the assessment of the sensitivity of our causal estimates to potential violations of the unconfoundedness assumption [42,43,44].

This step enabled us to understand the stability and reliability of our causal discoveries.

## 3. Results

### 3.1. Key Factors Influencing T. japonicus Density

The top nine factors are msl-0, Ozone_sum, Month, msl-4, Height, sp-0, sp-4, f10.7_index, and N3M20.

**Figure 2:**
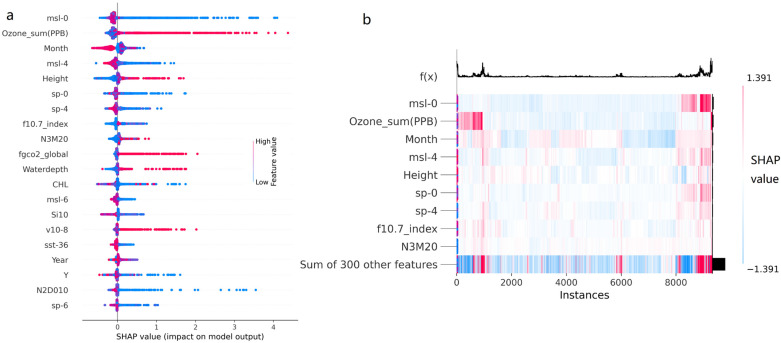
(**a**) Distribution of feature values and SHAP values for the top factors, where red indicates a high feature value, and blue indicates a low feature value. (**b**) Clustering distribution of SHAP values for the training set samples in the model, where in the function f(x), the base value is 1.0647. The significance of the impact is divided by 5% of the base value; that is, a significant impact is considered if |SHAP_value| > 0.0532; otherwise, the impact is considered insignificant. The training set samples consist of 9327 samples randomly drawn from the total samples at a ratio of 70% before modeling.

The factor msl-0 refers to the pressure of the atmosphere on the surface of the Earth adjusted to the height of the mean sea level without any lag effect. As shown in Figure 2a,b, the positive impact predominantly comes from the low values of this factor, with a sample proportion of 12.73% for SHAP value > 0.0532. The negative impact mainly comes from high values and a small portion of low values, with a sample proportion of approximately 82.64% for SHAP_value < −0.0532.

The factor Ozone_sum refers to the ozone concentration measured at the Summit weather station (Summit, Greenland) in Figure 2a,b, in units of PPB. When the value is high, it has a very significant positive impact on the model, with a sample proportion of 13.25% for positive significant impacts. When the value is low, it primarily has a very low negative impact, with a sample proportion of 73.83% for negative significant impacts (Figure 2).

The factor Month refers to the month of the survey. All negative impacts come from high values (winter months), with a sample proportion of 36.31%. Positive impacts are distributed at both low and high values, with a sample proportion of 47.50% (Figure 2).

The factor msl-4 refers to the mean sea level pressure with a lag effect of 4 days in Figure 2a,b. The positive impacts predominantly come from low values of this factor, with a sample proportion of 19.79% for positive significant impacts. The negative impacts mainly come from high values and a small portion of low values, with a sample proportion of 44.32% for negative significant impacts (Figure 2). The effect of this factor is similar to msl-0, but the impact strength (SHAP value) is relatively smaller.

The factor Height refers to the effective vertical range beneath the surface of the ocean water body detected by sonar. As shown in Figure 2a,b, positive impacts primarily come from high values of this factor, with a sample proportion of 15.24% for positive significant impacts. The negative impacts predominantly come from low values of this factor, with a sample proportion of 32.24% for negative significant impacts (Figure 2).

The factor sp-0 refers to the pressure of the atmosphere on the surface of land, sea, and inland water without any lag effect in Figure 2a,b. The positive impacts predominantly come from low values of this factor, with a sample proportion of 13.91% for positive significant impacts. The negative impacts primarily come from a small portion of high values and low values, with a sample proportion of 25.37% for negative significant impacts (Figure 2).

The factor sp-4 refers to the surface pressure with a lag effect of 4 days in Figure 2a,b. The positive impacts mainly come from low values of this factor, with a sample proportion of 20.65% for positive significant impacts. The negative impacts primarily come from a small portion of high values and low values, with a sample proportion of 30.25% for negative significant impacts. The effect of this factor is similar to sp-0, but the impact strength (shap value) is relatively smaller (Figure 2).

The factor f10.7_index refers to the F10.7 solar flux index, a measure of microwave solar emissions at a wavelength of 10.7 cm or 2800 MHz in Figure 2a,b. The positive impacts on the model primarily come from a small portion of high values and low values, with a sample proportion of 16.29% for positive significant impacts. The negative impacts predominantly come from the low values of this factor, with a sample proportion of 22.37% for negative significant impacts (Figure 2).

The factor N3M20 refers to the nitrate concentration at a water depth of 20 m in Figure 2a,b. The positive impacts on the model primarily come from some high values and a small portion of low values, with a sample proportion of 15.26% for positive significant impacts. The negative impacts predominantly come from low values of this factor, with a sample proportion of 14.71% for negative significant impacts (Figure 2).

### 3.2. A Complex Causal Network Revealed

**Figure 3:**
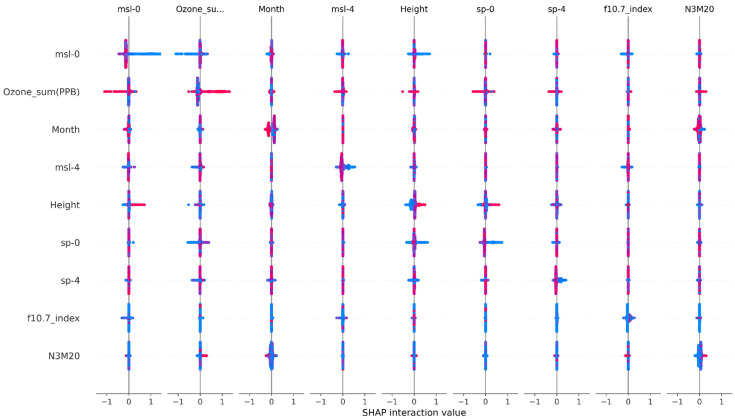
Interactive relationships among the top 9 factors, where red indicates a high feature value, and blue indicates a low feature value.

Interactive relationships among the key factors are shown in Figure 3. Interactions exist between msl-0 and Ozone_sum (PPB), Month, msl-4, Height, and f10.7_index. Similarly, Ozone_sum (PPB) interacts with msl-0, msl-4, Height, sp-0, sp-4, and N3M20, while Month interacts with msl-0, Ozone_sum (PPB), and N3M20. Msl-4 has relationships with msl-0, Ozone_sum (PPB), Height, and f10.7_index, and Height interacts with msl-0, Ozone_sum (PPB), sp-0, and sp-4. Furthermore, sp-0 has interactions with Ozone_sum (PPB), Height, and sp-4, while sp-4 interacts with msl-0, Ozone_sum (PPB), Month, Height, and sp-0. The f10.7 index factor interacts with msl-0 and msl-4, and N3M20 has interactions with Ozone_sum (PPB), Month, Height, sp-0, sp-4, and f10.7_index.

By integrating these interactions, the causality among the top 9 factors is refutatively validated, as shown in Table 1. The causality hypothesis between the control group factor, Random, and Biomass_density is refuted (*p*_value < 0.05), while the causality hypotheses of each factor in the Treatment Group are accepted (*p*_value > 0.05). However, the causality impact varies. In the New effect, the negative impact of the factor Month is the highest. The factor sp-0 has the highest positive impact, although its causality hypothesis is unstable. The causality hypothesis of the factor Ozone_sum is the most stable, but its impact is minimal.

**Figure 4:**
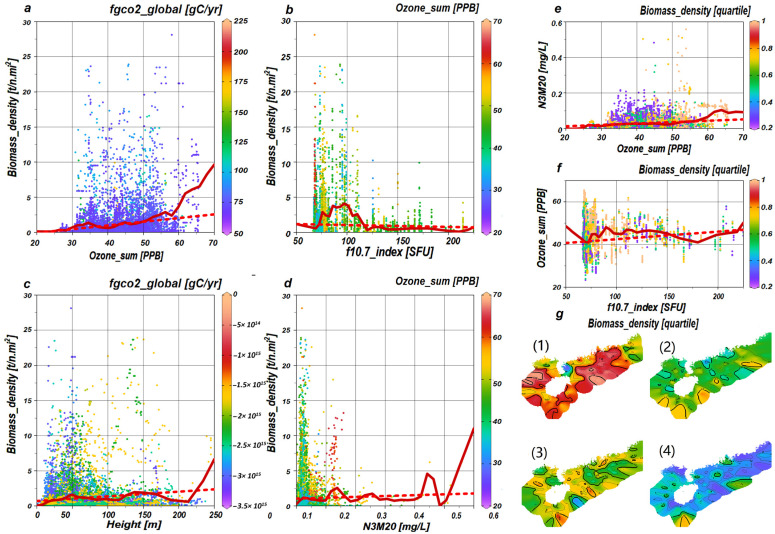
Visualization and succinct analysis of variables. The distribution of sample points for Ozone_sum, F10.7_index, Height, N3M20, and Biomass_density is displayed in panels (**a**–**d**), with the color coding corresponding to interaction variables. Panels (**e**,**f**) illustrate the distribution of sample points for the interactive relationships of Ozone_sum and N3M20 with F10.7_index and Ozone_sum, the color scheme of which is divided into quartiles based on Biomass_density. Panel (**g**) portrays the quartile distribution of Biomass_density, with sections (1)–(4) respectively demarcating the 0–25%, 25–50%, 50–75%, and 75–100% ranges. In panels (**a**–**f**), least-squares lines are denoted by red dotted lines, whereas solid red lines delineate the piece-wise linear least-squares lines.

Each variable manifests a distinct trend, as denoted by the dotted lines, in Figure 4a–f. For example, Ozone_sum reveals a positive trajectory, whereas F10.7_index displays a negative one. Despite these trends, Ozone_sum (Figure 4a) inclines towards an ascending curve, whereas F10.7_index presents a wave-like pattern that crests at 97 SFU (Figure 4b). In the context of interactive relationships, the positive trend of Ozone_sum relative to N3M20 is evident (Figure 4e), and the influence of F10.7_index on Ozone_sum is characterized by a wave-like trend (Figure 4f).

### 3.3. The Relevance of Ozone to Our Research Environment

**Figure 5:**
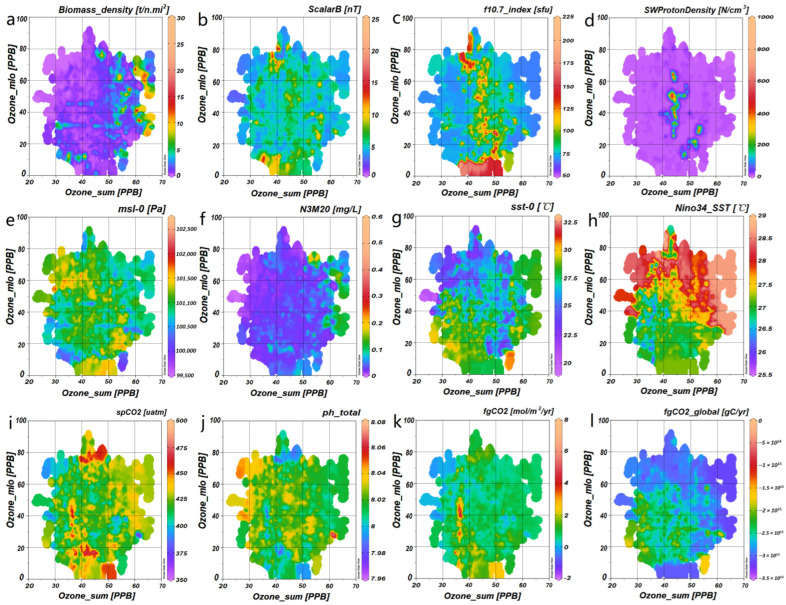
Presents the distribution spectra of correlated variables for Ozone at both high (SUM) and low (MLO) latitudes: (**a**) illustrates the distribution spectra of Ozone (MLO) and Ozone (SUM) in relation to the biomass density of *T. japonicus*; (**b**–**d**) correspond respectively to the distribution spectra of space weather factors such as Scalar B, the f10.7 index, and Solar Wind Proton Density; (**e**–**h**) represent the distribution spectra for climatic and aquatic environmental factors, such as msl_0, N3M20, sst_0, and Nino3.4_SST; (**i**–**l**) depict the distribution spectra for the main surface ocean carbon dioxide factors, such as spCO_2_, total pH, fgCO_2_, and global fgCO_2_. The color bar on the right side of each subfigure indicates the actual numerical range of the factor displayed at the top of the corresponding subfigure, with cool tones representing low values and warm tones denoting high values. The spatial patterns vary significantly across different factor categories. For instance, (1) space weather factors predominantly exhibit vertical banding anomalies; (2) climatic and aquatic environmental factors are characterized by horizontal or patchy distributions; (3) sea surface CO_2_ factors display both vertical banding and patchy patterns.

Ozone_SUM and Ozone_MLO serve as representative parameters for monitoring ozone concentrations at high and low latitudes, respectively. These variables significantly influence the biomass density of *T. japonicus* (Figure 5a) and are intricately and variably associated with factors such as the climatic conditions (Figure 5e–h), the carbon cycle (Figure 5i–l), and the space environment (Figure 5b–d).

For example, bursts in the f10.7 index and Solar Wind Proton Density typically cluster within the 35–55 PPB range of Ozone_SUM, whereas the Ozone_MLO during these bursts exhibits a broader distribution, ranging from 10 to 80 PPB (Figure 5c,d). The high-temperature regions of Nino3.4_SST are concentrated between 22 and 90 PPB of Ozone_MLO, with a wider distribution for Ozone_SUM. Conversely, the low-temperature regions are primarily found between 28 and 42 PPB of Ozone_SUM, again with a broader distribution for Ozone_MLO (Figure 5h). Lastly, the global variable, fgCO_2__global, demonstrates a trend of inverse correlation with ozone (Figure 5l).

## 4. Discussion

**Figure 6:**
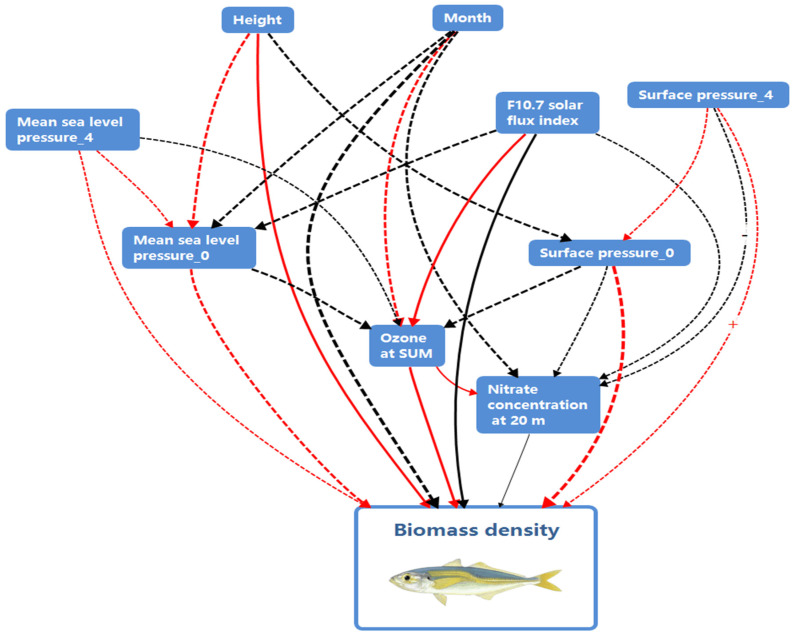
Schematic diagram of the primary causal relationship network based on the XGBoost model. Three types of markings in the figure require explanation: (1) Solid lines indicate relatively stable causal relationships, while dashed lines represent unstable relationships. (2) Line colors: Red denotes a positive influence, whereas black signifies a negative influence. (3) Line thickness: Thicker lines indicate a relatively stronger influence, while thinner lines represent weaker effects. It should be noted that the universality of the causal relationships illustrated here requires further validation. The figure is for reference only.

This study, through an integrated analysis of multi-scale environmental factors and jack mackerel biology, reveals that its population density is regulated by trans-oceanic biogeochemical cascades: The Arctic ozone concentration (Ozone_sum) affects phytoplankton productivity via ultraviolet radiation intensity, thereby limiting the key food supply for juveniles [45]; solar activity (F10.7_index) perturbs the geomagnetic field, disrupting magnetic navigation during adult seasonal migration [46]; while 20 m nitrate concentration (N3M20) drives a biphasic secondary productivity response—moderate enrichment promotes the diatom–copepod food chain, whereas exceeding the threshold triggers algal blooms and stoichiometric imbalance [47]. Climate-driven mixed-layer-depth changes restrict the diel vertical migration range of fish [32], trapping schools between deep oxygen-deficient zones and food-poor shallow layers. The results underscore the correlations among multiple climatic and environmental features. Among the most influential factors, sea-level air pressure (msl-0 and msl-4), surface air pressure (sp-0 and sp-4), ozone concentration (Ozone_sum), nitrate concentration at 20 m depth (N3M20), and the solar radio flux at 10.7 cm (f10.7_index) reflect a fascinating interdependency, all of which are directly or indirectly associated with the acoustic density distribution of *T. japonicus*. Based on these comprehensive results, a causal chain diagram (Figure 6) is presented to briefly illustrate the potential interrelationships among these factors. Ozone_sum, the F10.7 solar flux index, and N3M20 emerge as pivotal hubs within this causal network, serving as crucial nodes that maintain relatively stable relationships.

The month (Month) was identified as a significant driver influencing several features. This association likely mirrors seasonal variations in climatic conditions, sea temperature, and nutrient supply, possibly triggering fluctuations in fish populations [48]. The links observed among these parameters corroborate the complexity of marine ecosystems, where seasonal fluctuations broadly impact various biological and environmental aspects [49]. These relationships align with previous research, which found that seasonal factors play a critical role in species distribution [50], particularly in marine organisms.

The interaction between msl_0, msl_4, sp_0, and sp_4, the climatic and oceanic environment factors, might imply their complexity in impacting the stability of marine ecosystems and the distribution of organisms. Under the backdrop of global changes, the effects of climate change might not be limited to the alteration of a single environmental variable but encompass the interaction of multiple environmental variables [51].

The association with Ozone_sum may indicate the ecological impacts of global environmental factors at the regional scale. Variations in ozone concentration could affect the intensity of sunlight and UV radiation at the ocean surface, thereby impacting marine ecosystems, including the survival and distribution of fish species [52]. Interestingly, it is important to highlight that Ozone_mlo, despite being at the same latitude as Biomass_density, has a lower impact than Ozone_sum, found at higher latitudes. This underscores the far-reaching influence of Arctic ozone concentrations on a global scale [10,11,53].

A fundamental attribute of this study is the revelation of a complex causal network among the primary features. The interactions between atmospheric pressure, ozone concentration, solar radio flux, and nitrate concentration unveil the influence of atmospheric and marine processes on fish distribution. Indeed, previous research has pointed out the potential impact of atmospheric pressure on sea temperature, subsequently affecting marine organisms [54]. The causal relationship between the f10.7_index, N3M20, and fish distribution density might reflect the influence of solar activity and nutrient salt concentration on marine organism distribution. Variations in solar activity might affect Earth’s magnetic field, subsequently influencing the migration and distribution of certain magnetically sensitive fish species [46]. The role of nitrate concentration mirrors the dynamic of nutrients in marine ecosystems, influencing primary productivity and hence the food supply to fish [55]. Moderate nitrate loading enhances diatom-derived copepod production [45]. Conversely, hypereutrophication triggers stoichiometric mismatches, establishing a competitive bottleneck that decouples nitrate availability from fish production [56]. This dual-threshold mechanism (nutrient limitation vs. stability loss) reveals that fisheries’ management must regulate coastal nitrate sinks not only beyond simple concentration targets but also considering phytoplankton composition and zooplanktivore competition dynamics [45,47,56].

Although anthropogenic factors, particularly maritime activities and fishing operations, did not manifest a strong causality in the model, their possible impacts should not be overlooked. Human activities exert multifaceted impacts on marine ecosystems, including direct influences (e.g., overfishing and habitat destruction) and indirect impacts (e.g., climate change and ocean acidification due to greenhouse gas emissions). Data on maritime activities could represent a potential source of pressure, and fishing could directly impact fish populations [57].

Meanwhile, even though global carbon sink-related factors such as sea surface CO_2_ partial pressure did not show significant causal relationships in this study, their roles in long-term trends and global environmental changes and their potential ecological impacts, such as ocean acidification, warrant continuous attention [58,59].

## 5. Conclusions

This study reveals that the causally driven relationships between ozone concentration (Ozone_sum), solar activity (F10.7_index), nitrate dynamics (N3M20), and water depth (Height) necessitate transformative shifts in fisheries’ governance frameworks. From a management perspective, our findings advocate for establishing dynamic quota systems that incorporate real-time monitoring of these remote drivers. Specifically, when elevated ozone concentrations (>40 PPB) coincide with a low solar radiation flux (F10.7 index < 90 SFU), biomass density often exhibits significant peaks (Figure 4b,f), suggesting that this period may be more conducive to the natural replenishment of population resources. Therefore, it is advisable to implement preventive fishing control measures in a timely manner to avoid resource depletion caused by overfishing during population recovery.

Conservation efforts must prioritize safeguarding critical vertical migration corridors in areas where the sonar-measured habitat depth (Height) falls below 40 m, as compressed habitats intensify predation pressure and resource competition under climatic stressors. Critically, the way that ozone-mediated ultraviolet fluctuations significantly impact larval survival implies that marine protected areas should integrate UV-shielding coastal features (e.g., mangrove forests, turbidity plumes) into nursery habitat designs.

For predictive applications, changes in Greenland ozone impact plankton prey availability, indirectly shaping jack mackerel (*T. japonicus*) abundance (Figure 4d,e) and enabling optimized fishing vessel deployment. Embedding these teleconnections into ecosystem models (e.g., Atlantis, Ecopath) would rectify current underestimations of cross-latitudinal climate couplings in stock assessments. Failure to operationalize these discoveries risks mismanaging this near-threshold species amid accelerating polar–tropical feedback loops.

## Figures and Tables

**Table 1:** Refutation of causal reasoning.

Group	Factor	Estimated Effect	Data Subset Refutation		Random Common Cause Refutation	
			New Effect	*p*_Value	New Effect	*p*_Value
Treatment Group	msl-0	0.01	0.10	0.26	0.09	0.16
	**Ozone_sum**	0.09	0.09	**0.90**	0.08	**0.96**
	msl-4	−0.18	0.01	0.22	0.03	0.20
	sp-0	2.68	0.23	0.08	0.29	0.06
	sp-4	0.13	0.06	0.38	0.06	0.54
	**F10.7_index**	−0.20	**−0.11**	**0.66**	**−0.18**	**0.70**
	Month	−0.10	**−0.32**	0.16	**−0.34**	0.08
	**Height**	0.17	0.15	**0.74**	0.15	**0.94**
	**N3M20**	0.03	−0.01	**0.70**	−0.02	**0.58**
Control Group	Random	−0.26	0.01	0.01 *	0.01	0.00 *

Note: Asterisk (*) indicates significance but unstable differences at *p* < 0.01. **Bold** type indicates the variable has a close relationship with species density.

## Data Availability

The original contributions presented in the study are included in the article. The data presented in this study are available on request from the corresponding author.

## Supplementary Materials

The following supporting information can be downloaded at: https://www.mdpi.com/article/10.3390/biology14070895/s1. Tables S1 and S2: Biomass density based on hydroacoustics and catch; Tables S3–S5: Spatiotemporal variables; Table S6: Globle variables; Table S7: Model Selection; Figure S1: Collinearity of key factors; Table S8: Summary of Variables.
