# Supplementary material for: Exploring the Global and Regional Factors Influencing the Density of Trachurus japonicus in the South China Sea
Source: Biology (Basel). 2025 Jul 21;14(7):895. doi: 10.3390/biology14070895 (PMC12292112; doi:10.3390/biology14070895)
Supplement: Supplementary file 1 [file biology-14-00895-s001.zip › biology-3681723-supplementary.pdf]

Contents

1. Supplementary Methods, Biomass density based on hydroacoustics and catch (Tabels S1 and S2)

2. Supplementary Methodology, Spatiotemporal variables(Tabels S3-S5)

3. Supplementary Methodology, Globle variables(Tabel S6)

4. Supplementary Methodology, Model Selection(Tabel S7)

5. Supplementary Methodology, Collinearity of key factors (Figure S1)

6. Supplementary Methodology, Summary of Variables (Tabel S8)

Supplementary Methodology

1. Biomass Density Based on Hydroacoustic Data and Catch Information

The estimation of fish biomass density was achieved through the conversion and subsequent analysis of hydroacoustic survey data.

Initially, hydroacoustic data were gathered utilizing a portable Simrad EY60 split-beam echo sounder, which operated at frequencies of 70 and 120 kHz (refer to Table 1). The frequency of 120 kHz served as the reference for biomass estimation, with 70 kHz acting as a supplementary reference frequency, as its resolution is ill-suited for smaller, individual targets. Prior to each survey, the echo sounder was calibrated. Following data collection, the Echoview Fishery Acoustic Data Processing System (version 12.0) was employed for data analysis. Fish schools were manually identified within the dataset. All hydroacoustic data underwent rigorous review to exclude targets that were not marine organisms, such as air bubbles, instrumental noise, and zooplankton patches. The analysis focused on the water layer between 5 m and 300 m, with an analysis unit set at 3 nmi and an integration threshold set at –80 dB. The function of "background noise removal" was utilized in later stages to eliminate background noise from specific areas.

Table S1. Main technical parameters of Simrad EY60

Technical parameter	Value		Unit
	70 kHz energy converter	120 kHz energy converter	
Transmitting power	800	500	W
Pulse width	0.512	0.512	ms
Detection range	1000	800	m
Gain of the energy converter	27.00	27	dB
Longitudinal beam angle 3dB beam width	7.00	7.00	degree

Horizontal beam angle	7.00	7.00	degree
3dB beam width			
Absorption coefficient	0.018	0.045	dB·m^{-2}
Sound velocity	1535	1535	m/s
Beam equivalent solid angle	-21	-21	dB

Catch data were procured from 99 distinct locations utilizing a single boat bottom otter trawl featuring a circumference of 80.80 m and a mesh size of 20 cm along the net's leading edge. The net measured 60.54 m in total length, with a mesh size of 39 mm. Approximately 60 minutes were required to collect samples from each location, following which the total mass and sample count were calculated. Key parameters of the catches included species name, minimum and maximum body length, minimum and maximum weight, total quantity, and total weight. Moreover, the length-frequency distributions for the main species were determined. Catch data and nutrient salts, originally collected at station level, were expanded to regional data using Ordinary Kriging interpolation, and subsequently extracted according to the coordinates of NASC. The analysis unit of biomass density mirrored that of NASC, both set at 3 nmi. The interpolation grid featured cell dimensions of 0.0286° by 0.0286°.

Biomass density (tonnes/n.mi2) for each fish species within the surveyed area was computed using the subsequent formulae:

$$Biomass\_density(tonnes/n.mi^2) = \frac{\%contribution}{100} \times \frac{NASC}{4\pi\bar{\sigma}} \times \frac{W}{1000} \quad \text{(S1)}$$

$$\bar{\sigma} = \sum_{all\ species}\left(\frac{\%contribution}{100} \times 10^{\frac{TS}{10}}\right) \quad \text{(S2)}$$

$$\mathrm{TS} = 20 \cdot \log_{10} L + b_{20} \quad \text{(S3)}$$

$$\mathrm{L} = (\mathrm{w_i} \times 10^3/a)^{1/b} \quad \text{(S4)}$$

In a similar manner, the biomass density of *T. japonicus* was computed based on formula

(S1). This calculation was grounded on the proportion of catches for each group or population at a given position, in combination with the mean weight of the catches (W, kg), and the $\bar{\sigma}$ of the group or population at the trawl position. In the aforementioned formulae, the Nautical Area Scattering Coefficient (NASC, $m^2/n.mi^2$), $\bar{\sigma}$, and Target Strength (TS, dB) constitute the hydroacoustic parameters; L represents the body length of the fish (cm); b_{20} is the compensation coefficient corresponding to TS if the coefficient of $\log_{10} L$ is 20. The value of b_{20} was derived using formula (S3), as outlined in the Acoustic Assessment on Fishery Resources in the northern South China Sea spanning the years 1997 to 1999 (refer to Table S2).

Table S2. Hydroacoustic Information of *T. japonicus*

Species	No.	b_{20}	a	b	Habitus
T. japonicus	Biomass_density	-72.5	0.0106	3.1000	Middle and upper layers

2. Spatiotemporal Variables

The spatiotemporal variables encompassed the water environment, climate, surface-ocean carbon dioxide, and AIS (Automatic Identification System) fishing.

(1) The water environment incorporated standard variables like nutrients and CTD. The AML Plus X was utilized for determining environmental factors impacting water quality, which comprised water temperature, salinity, water depth, and additional indices such as nutrients and transparency. Nutrient samples were collected at depths of 0 m, 10 m, and 20 m. The environmental factors influencing water quality were categorized into primary and derived factors. The mixed surface layer's primary characteristics were surface salinity (SS, ppt) and surface temperature (ST, °C) at 2 m. In contrast, the bottom cold water layer's primary characteristics were bottom salinity (BS, ppt) and bottom temperature (BT, °C) at 2 m above the bottom, water depth (WD, m), longitude (X, °), latitude (Y, °), time (24 h), year, month, the full moon ratio (moon), and chlorophyll concentration (CHL, mg/m3). Derived factors were computed using the primary factors and included salinity (DS, ppt) and temperature difference (DT, °C) between the surface and the bottom coldwater layers, as well as the difference in NO_2 concentration at 0 m and 10 m (N2-d010, mg/L) and several

other parameters listed in Table 1. This study classified environmental factors influencing water quality into five categories: 1) Spatial metadata; 2) Time metadata; 3) Hydroacoustic information; 4) Primary water environmental factors (total 20); and 5) Recalculate water environmental factors, including all derived factors (total 17). All factors were resampled according to the longitude and latitude of NASC coordinate points. The environmental factors (totaling 46) are also synchronization factors, as they correspond one-to-one with the density samples of the group or population at the same time and space (refer to Table S3).

Table S3. Listing and Grouping of Water Environmental Factors

**Group**	**Factor (abbreviation)**	**Name**	**Unit**	**Note**
Spatial metadata	Water depth	water depth	m	
	X	longitude	°E	
	Y	latitude	°N	
Time metadata	Year	Year: 2014 –2020		
	Month	Month: 1,2,3,4,5,6,7,8,9,10,11		
	Time	Time (24 h): 0.00–23.99		
	Moon	Phase of the moon:0–1		
Hydroacoustic information	Height	Height of the 3D region (Depth_max-Depth_min)	m	
	Ping	Number of pulses received per 3 nautical miles, reflecting the speed of the ship		
Primary water environmental factors	SS	surface salinity	ppt	Surface
	BS	bottom salinity	ppt	Bottom
	ST	surface temperature	° C	Surface
	BT	bottom temperature	° C	Bottom
	CHL	chlorophyll concentration	mg/m^3	Surface

	N2M0	NO_2^- 0 m concentration	mg/L	Surface
	N2M10	NO_2^- 10 m concentration	mg/L	Surface
	N2M20	NO_2^- 20 m concentration	mg/L	Bottom
	N3M0	NO_3^- 0 m concentration	mg/L	Surface
	N3M10	NO_3^- 10 m concentration	mg/L	Surface
	N3M20	NO_3^- 20 m concentration	mg/L	Bottom
	N4M0	NH_4^+ 0 m concentration	mg/L	Surface
	N4M10	NH_4^+ 10 m concentration	mg/L	Surface
	N4M20	NH_4^+ 20 m concentration	mg/L	Bottom
	P0	PO_4^{3-} 0 m concentration	mg/L	Surface
	P10	PO_4^{3-} 10 m concentration	mg/L	Surface
	P20	PO_4^{3-} 20 m concentration	mg/L	Bottom
	Si0	SiO_3^{2-} 0 m concentration	mg/L	Surface
	Si10	SiO_3^{2-} 10 m concentration	mg/L	Surface
	Si20	SiO_3^{2-} 20 m concentration	mg/L	Bottom
Recalculate water environmental factors	DS	salinity difference between surface and bottom layers	ppt	
	DT	temperature difference between surface and bottom layers	°C	
	N2D010	concentration difference between NO_2^- 0 m and 10 m	mg/L	
	N2D020	concentration difference between NO_2^- 0 m and 20 m	mg/L	
	N2D1020	concentration difference between NO_2^- 10 m and 20 m	mg/L	
	N3D010	concentration difference between NO_3^- 0 m and 10 m	mg/L	
	N3D020	concentration difference	mg/L	

	between NO_3^- 0 m and 20 m	
N3D1020	concentration difference between NO_3^- 10 m and 20 m	mg/L
N4D010	concentration difference between NH_4^+ 0 m and 10 m	mg/L
N4D020	concentration difference between NH_4^+ 0 m and 20 m	mg/L
N4D1020	concentration difference between NH_4^+ 10 m and 20 m	mg/L
P010	concentration difference between PO_4^{3-} 0 m and 10 m	mg/L
P020	concentration difference between PO_4^{3-} 0 m and 20 m	mg/L
P1020	concentration difference between PO_4^{3-} 10 m and 20 m	mg/L
Si010	concentration difference between SiO_3^{2-} 0 m and 10 m	mg/L
Si020	concentration difference between SiO_3^{2-} 0 m and 20 m	mg/L
Si1020	concentration difference between SiO_3^{2-} 10 m and 20 m	mg/L

(2) Regarding climate extraction, the location was determined by NASC coordinates, and the time was based on the hour closest to NASC time. The factors were separated into 8 categories, including a 10 m u-component of wind and a 2 m temperature component (refer to Table S4). In the abbreviated representations of factors, "-n" symbolized the number of days that climatic factors were sampled prior to the sampling of the group density of

Scombridae fish, indicating the time-lag effect period. Furthermore, the value of "n" ranged from 0, 2, 4, …, to 40, where n = 0 was considered a synchronous factor corresponding one-to-one with the group or population density sampled at the same time and space, denoted as "-0". For instance, "u10–0" represented a synchronous climate factor "10 m u-component of wind", while "sst-12" denoted a climatic factor "sea surface temperature" sampled 12 days before the group density of Scombridae fish was determined. Climate variables were downloaded from https://cds.climate.copernicus.eu/cdsapp#!/dataset/reanalysis-era5-single-levels?tab=overview. This study selected climate variable lag times of 0-40 days (at 2-day intervals, totaling 21 time points) based on three scientific rationales.1) Ecological Response Window: The 40-day upper limit covers the critical period of phytoplankton-fish trophic cascades (plankton response ~10-20 days; chub mackerel feeding migration lagging 3-4 weeks);2) Causal Robustness: Random common cause refutation tests confirmed stable causal relationships for lagged variables;3) Resolution Optimization: Interval design avoids redundancy while capturing nonlinear responses.

Table S4. Principal Climatic Factors

**Factor (abbreviation)**	**Name**	**Units**	**Description**
u10	10 m u-component of wind	m/s	This parameter is the eastward component of the 10 m wind, and it is the horizontal speed of air moving toward the east, at a height ten meters above the surface of the Earth, in meters per second.
v10	10 m v-component of wind	m/s	This parameter is the northward component of the 10 m wind, and it is the horizontal speed of air moving toward the north, at a height ten meters above the surface of the Earth, in meters per second.
d2m	2 m dewpoint	°C	This parameter is the temperature to which the air, at 2 meters above the surface of the Earth, would have to be cooled for saturation to occur.

	temperature		
t2m	2 m temperature	°C	This parameter is the air temperature at 2 m above the surface of land, sea, or inland waters.
msl	Mean sea level pressure	Pa	This parameter is the pressure (force per unit area) of the atmosphere on the surface of the Earth adjusted to the height of the mean sea level.
sst	Sea surface temperature	°C	This parameter (SST) is the temperature of seawater near the surface.
sp	Surface pressure	Pa	This parameter is the pressure (force per unit area) of the atmosphere at the surface of land, sea, and inland water.
tp	Total precipitation	m	This parameter is the accumulated liquid and frozen water, comprising rain and snow, that falls to the Earth's surface.

(3) The surface-ocean carbon dioxide variables encompass ph_total, talk, sfco2, pco2,hco3, co3, co2, fgco2, and fgco2_global (refer to Table S5 for more details). This collection provides a globally gridded dataset of the surface ocean carbonate system, suitable for the study of ocean acidification over seasonal to decadal periods (version 2023). The full marine carbonate system is derived from machine learning predictions of Total Alkalinity (TA) and the fugacity of carbon dioxide (fCO_2). The dataset can be accessed from https://www.ncei.noaa.gov/metadata/geoportal/rest/metadata/item/gov.noaa.nodc%3A0220059/html.

Table S5. Parameters Pertaining to Surface-Ocean Carbon Dioxide

**Factor (abbreviation)**	**Units**	**Description**

ph_total	-log([H+])	Surface ocean pH on the total scale.
talk	umol/kg	Total alkalinity estimated with an ensemble of SVR models (scikit-learn).
sfco2	uatm	Surface dfco2 predicted with a two-step cluster-regression approach (pco2atm added back after estimation).
spco2	uatm	Surface_partial_pressure_of_carbon_dioxide_in_sea_water.
hco3	umol/kg	
co3	umol/kg	
co2	umol/kg	
fgco2	$mol/m^2/yr$	Sea-air flux calculated using the SeaFlux dataset. kw is calculated using ERA5, JRA55 and NCEP1 winds. Solubility is calculated using the Weiss (1974) parameterization. SST and ice are from the OISSTv2 data product. Salinity is from EN4.2.1.
fgco2_global	gC/yr	Spatially integrated sea-air CO_2 flux in gC/yr.

(4) The global datasets of AIS-based fishing effort and vessel presence were obtained from https://globalfishingwatch.org/data-download/datasets/public-fishing-effort. Fishing vessels are discerned through a neural network classifier, vessel registry databases, and manual evaluation conducted by the Global Fishing Watch (GFW) and regional specialists. Data are arranged into grid cells measuring 0.01 degrees on each side, with time units expressed in hours. The total time is calculated by attributing the interval between each AIS detection to the previous position, followed by summing all positions within each grid cell.

To examine the influence of grid cell dimensions on fish distribution, we established multiple radial scales, including 0.025, 0.05, 0.075, 0.1, 0.125, 0.15, 0.175, 0.2, 0.225, 0.25, 0.275, 0.3, 0.325, 0.35, 0.375, 0.4, 0.425, 0.45, 0.475, 0.5, 0.6, 0.7, 0.8, 0.9, and 1.0. For each timescale, the values of various parameters within the sampling point's radial range are

aggregated to yield new variables. For instance, hours_0.025 denotes the cumulative sailing time within a 0.025° radius, fishing_hours_0.025 stands for the cumulative fishing time within a 0.025° radius, and mmsi_present_0.025 represents the number of vessels within a 0.025° radius.

3. Globle variables

Global variables consist of the Nino index, surface ozone, and geomagnetic field-linked space weather variables.

(1) Nino34 (SST) refers to the Sea Surface Temperature in the central equatorial Pacific (Niño 3.4 region). Nino34(SSTA) is shorthand for Sea Surface Temperature Anomaly, indicating the deviation of the sea surface temperature in the central equatorial Pacific (Niño 3.4 region) from its long-term average. Positive anomalies usually signify El Niño events, whereas negative anomalies typically denote La Niña events. The associated data can be retrieved from https://origin.cpc.ncep.noaa.gov/data/indices/wksst8110.for.

(2) Ozone_sum and Ozone_mlo represent the surface ozone (O_3) concentration at the Summit weather station (Summit, Greenland, N72.59621°, W38.42201°) and Mauna Loa weather station (Mauna Loa, Hawaii, N19.53622°, W155.57632°), respectively. The units are in PPB, and all sample data are hourly averages recorded in situ. The data can be downloaded from https://gml.noaa.gov/dv/data/index.php.

(3) Space weather variables associated with the geomagnetic field primarily include Scalar B (nT), SW Proton Density (N/cm^3), Flow pressure, Kp_index, Dst_index (nT), and f10.7_index (see Table S6 for details). The data can be downloaded from https://omniweb.gsfc.nasa.gov/form/dx1.html. The 10.7cm Solar Flux reflects the integrated emission at a 10.7cm wavelength from all sources present on the solar disk, predominantly thermal in origin, and directly linked to the total amount of plasma entrapped in the magnetic fields overlaying active regions.

Table S6. Space Weather Variables Pertinent to Geomagnetism

Factor (abbreviation)	Units	Description

Scalar B	nT	Field magnitude average, field vector, |<B>|.
SW Proton Density	N/cm^3	Solar wind proton density.
Flow pressure	nPa	The dynamic pressure of the solar wind, determined by the density and speed of the solar wind.
Kp_index	1	A parameter measuring geomagnetic activity, 3-hr Kp index from GFZ, Potsdam.
Dst_index	nT	A parameter measuring disturbances in the geomagnetic field from Kyoto, mainly used to assess the intensity of geomagnetic storms.
f10.7_index	SFU	A daily index measuring solar radio flux. The f10.7 index shows significant variations during the solar activity cycle.

4. Model Selection

We employed four algorithms for modeling and discovered that the machine learning algorithm XGBoost exhibits superior performance and accelerated modeling speed, aligning with our expectations.

Table S7. Comparative Analysis of Model Performance

	Linear Regression	Lasso	Random Forest	XGBoost
MSE	3.10	6.03	0.58	**0.34**
R^2	0.49	0.14	0.90	**0.94**

5. Collinearity of key factors

Although the correlation coefficients between msl-4 and sp-4, and between msl-0 and sp-0, are relatively high, we retained these factors because Mean Sea Level Pressure and Surface Pressure represent distinct parameters with unique scientific significance.

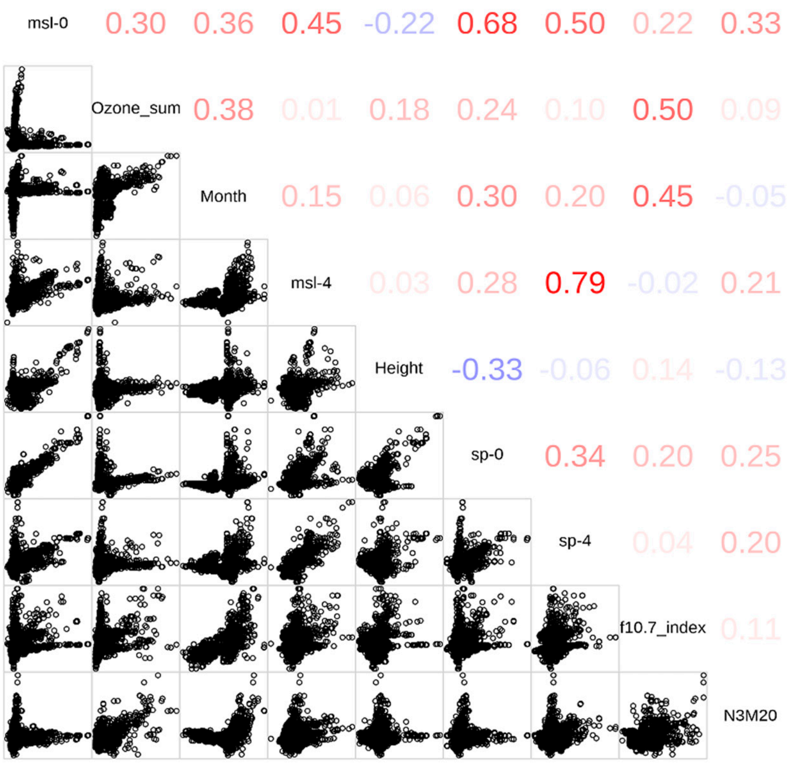

Figure S1. Spearman correlation matrix of key factors

6. Summary of Variables

Table S8. Comprehensive List of Variables Used for Modeling

Statistical classification of Variables	Ecological classification of variables	Number of Variables	Variable name
Target variable	Fish biomass density	1	Biomass density of *T.japonicus*
Feature variables （Treatment）	Actual water environment and geographic information, etc	46	Height, Ping, Y, X, Time, Year, Month, Moon, Waterdepth, ST, BT, SS, BS, CHL, N2M0, N3M0, N4M0, P0, Si0, N2M10, N3M10, N4M10, P10, Si10, N2M20, N3M20, N4M20, P20, Si20, DT, DS, N2D010, N3D010, N4D010, P010, Si010, N2D020, N3D020, N4D020, P020, Si020, N2D1020, N3D1020, N4D1020, P1020, Si1020
	Climate	168	tp-0, sst-0, d2m-0, v10-0, sp-0, u10-0, msl-0, t2m-0, sp-2, d2m-2, t2m-2, msl-2, tp-2, u10-2, v10-2, sst-2, sp-4, d2m-4, t2m-4, msl-4, tp-4, u10-4, v10-4, sst-4, sp-6, d2m-6, t2m-6, msl-6, tp-6, u10-6, v10-6, sst-6, sp-8, d2m-8, t2m-8, msl-8, tp-8, u10-8, v10-8, sst-8, sp-10, d2m-10, t2m-10, msl-10, tp-10, u10-10, v10-10, sst-10, sp-12, d2m-12, t2m-12, msl-12, tp-12, u10-12, v10-12, sst-12, sp-14, d2m-14, t2m-14, msl-14, tp-14, u10-14, v10-14, sst-14, sp-16, d2m-16, t2m-16, msl-16, tp-16, u10-16, v10-16, sst-16, sp-18, d2m-18, t2m-18, msl-18, tp-18, u10-18, v10-18, sst-18, sp-20, d2m-20, t2m-20, msl-20, tp-20, u10-20, v10-20, sst-20, sp-22, d2m-22, t2m-22, msl-22, tp-22, u10-22, v10-22, sst-22, sp-24, d2m-24,

		t2m-24, msl-24, tp-24, u10-24, v10-24, sst-24, sp-26, d2m-26, t2m-26, msl-26, tp-26, u10-26, v10-26, sst-26, sp-28, d2m-28, t2m-28, msl-28, tp-28, u10-28, v10-28, sst-28, sp-30, d2m-30, t2m-30, msl-30, tp-30, u10-30, v10-30, sst-30, v10-32, sp-32, d2m-32, u10-32, sst-32, tp-32, msl-32, t2m-32, v10-34, sp-34, d2m-34, u10-34, sst-34, tp-34, msl-34, t2m-34, v10-36, sp-36, d2m-36, u10-36, sst-36, tp-36, msl-36, t2m-36, v10-38, sp-38, d2m-38, u10-38, sst-38, tp-38, msl-38, t2m-38, v10-40, sp-40, d2m-40, u10-40, sst-40, tp-40, msl-40, t2m-40
El Niño indices	2	Nino34(SST), Nino34(SSTA)
Ozone	2	Ozone_sum(PPB), Ozone_mlo(PPB)
Geomagnetic data	6	ScalarB(nT), SWProtonDensity(N/cm^3), Flowpressure, Kpindex, Dst-index(nT), f10.7_index
Surface-ocean carbon dioxide data	9	ph_total, talk, sfco2, spco2, hco3, co3, co2, fgco2, fgco2_global
AIS-based fishing effort and vessel presence	75	hours_0.025, fishing_hours_0.025, mmsi_present_0.025, hours_0.05, fishing_hours_0.05, mmsi_present_0.05, hours_0.075, fishing_hours_0.075, mmsi_present_0.075, hourst_0.1, fishing_hours_0.1, mmsi_present_0.1, hours_0.125, fishing_hours_0.125, mmsi_present_0.125, hours_0.15, fishing_hours_0.15, mmsi_present_0.15, hours_0.175, fishing_hours_0.175, mmsi_present_0.175, hours_0.2, fishing_hours_0.2, mmsi_present_0.2, hours_0.225, fishing_hours_0.225, mmsi_present_0.225, hours_0.25, fishing_hours_0.25, mmsi_present_0.25,

			hours_0.275, fishing_hours_0.275, mmsi_present_0.275, hours_0.3, fishing_hours_0.3, mmsi_present_0.3, hours_0.325, fishing_hours_0.325, mmsi_present_0.325, hours_0.35, fishing_hours_0.35, mmsi_present_0.35, hours_0.375, fishing_hours_0.375, mmsi_present_0.375, hours_0.4, fishing_hours_0.4, mmsi_present_0.4, hours_0.425, fishing_hours_0.425, mmsi_present_0.425, hours_0.45, fishing_hours_0.45, mmsi_present_0.45, hours_0.475, fishing_hours_0.475, mmsi_present_0.475, hours_0.5, fishing_hours_0.5, mmsi_present_0.5, hours_0.6, fishing_hours_0.6, mmsi_present_0.6, hours_0.7, fishing_hours_0.7, mmsi_present_0.7, hours_0.8, fishing_hours_0.8, mmsi_present_0.8, hours_0.9, fishing_hours_0.9, mmsi_present_0.9, hours_1.0, fishing_hours_1.0, mmsi_present_1.0
Feature variable (Blank control)	Meaningless data	1	Random
